# Impact of Eco-Friendly Flame-Retardant Water-Blown Rigid Polyurethane Foams Containing Recycled Polyols for Insulation Applications

**DOI:** 10.3390/polym18070856

**Published:** 2026-03-31

**Authors:** Mercedes Santiago-Calvo, Izotz Amundarain, José Luis Gómez-Alonso, Jesús Ballestero, Sixto Arnaiz, Esteban Cañibano, María-Teresa Fernández

**Affiliations:** 1 Foundation for Transport and Energy Research and Development (CIDAUT), Parque Tecnológico de Boecillo, 47151 Valladolid, Spain; estcan@cidaut.es (E.C.); maifer@cidaut.es (M.-T.F.); 2 GAIKER Technology Centre, Basque Research and Technology Alliance (BRTA), Parque Tecnológico de Bizkaia, Edificio 202, 48170 Zamudio, Spain; amundarain@gaiker.es (I.A.); gomez@gaiker.es (J.L.G.-A.); ballestero@gaiker.es (J.B.); arnaiz@gaiker.es (S.A.)

**Keywords:** polyurethane foam, recycled polyols, thermal conductivity, fire properties, halogen-free flame retardants

## Abstract

The need to reduce polyurethane (PU) foam waste has encouraged the development of sustainable foam formulations based on recycled raw materials and environmentally friendly additives, addressing both waste management and comparable foam properties to those based on fossil resources. In the present investigation, more sustainable water-blown rigid PU foams were investigated using recycled polyol and halogen-free flame retardants (FRs) for fire-resistant insulation applications. Two series of foam formulations were prepared: a first series with virgin polyol and the inclusion of a halogen-free FR additive (6 wt%) and a second series with recycled polyol (10% added respect to the total polyol) and halogen-free FR additives (6 wt%). Two types of FR were used: FR900, specifically identified as 3,9-Dimethyl-2,4,8,10-tetraoxa-3,9-diphosphaspiro[5.5]undecane-3,9-dioxide, in powder form with 24% phosphorus content and reactive polyol based FR140, an oligomeric ethyl ethylene phosphate, in liquid form with 19% phosphorus content. The density, cellular structure, aged thermal conductivity, dimensional and hydrolytic stability, fire properties, and mechanical properties were characterized for novel foamed systems. Rigid foamed materials with very low densities around 50 kg/m^3^ were obtained. On the one hand, the inclusion of FR900 into the PU formulation containing virgin polyol generated foam with the lowest thermal conductivity (36.10 mW/mK) due to the smaller open cell content (11.7%) and cell size reduction (433 microns). On the other hand, the inclusion of recycled polyol reduced the foam density by 6 kg/m^3^ (44.1 kg/m^3^), increased the cell size average (848 microns) and open cell content (15.1%), maintained thermal conductivity (38.73 mW/mK), slightly improved the fire properties, and worsened the mechanical properties in comparison with the PU reference containing only virgin polyol. The results obtained by the foam containing recycled polyol and 6% FR900 are remarkable, presenting an increase in density (50.3 kg/m^3^) and in open cell content (73%), but a very high reduction in cell size (465 microns) and thus a low value of thermal conductivity of 37.04 mW/mK with respect to the reference material containing recycled polyol. Moreover, this PU foam containing recycled polyol and FR900 offered improved fire resistance (148.2 kW/m^2^ of Maximum Average Rate of Heat Emission (MARHE), 179.1 kW/m^2^ of Maximum Heat Release Rate (HRRmax), and 24.6 MJ/m^2^ of Total Heat Release (THR)) and mechanical properties (6.97 MPa of Young’s modulus and 0.24 MPa of collapsed stress) for the construction sector. The inclusion of FR140 does not improve the properties of the foam system containing recycled polyol, mainly due to the deterioration of the cellular structure (in the open cell content and cell size).

## 1. Introduction

Polyurethane (PU) is one of the most versatile polymers, offering a wide variety of commercial applications. PUs can be classified mainly into foams and CASEs (Coatings, Adhesives, Sealants, Elastomers) [1]. Foams are further subdivided into flexible foams, such as those used in mattresses, car seats, or packaging; and rigid foams, which are generally used as insulation in buildings and for commercial and domestic refrigeration [2,3]. However, one of the main drawbacks to their great commercial success is the challenging management of the large amount of waste that is generated when the products including those foams reach their end of life [4]. Given the fact that PU foams are thermosetting materials, recycling them presents significant challenges, which have driven the exploration for sustainable strategies for their recovery and reincorporation into new products [5,6].

Currently, the most common destination of PU foam waste continues to be incineration or landfill, methods with a significant environmental impact [5,6,7,8,9]. Thus, recycling is the most efficient and economically viable alternative for managing this type of waste. PU foam recycling methods can be classified as mechanical, chemical, and energy recovery. Mechanical recycling involves crushing the material into fine particles for subsequent reincorporation without altering its chemical structure. This process is simple and low-cost, although the properties of the recovered product are often limited, which restricts its field of application [5,6]. Energy recovery, on the other hand, is based on the combustion of waste to harness its energy content, but it has significant disadvantages, such as the presence of FRs and the possible emission of toxic compounds such as hydrogen cyanide, carbon monoxide, and nitrogen oxides during thermal degradation [5,6]. Chemical recycling is the most sustainable option from both an environmental and economic perspective, as it allows the urethane bonds in thermosetting polymers to be broken under controlled conditions, thereby recovering reusable polyols as the main products [5,6,7,8,9]. Among the different methods used, glycolysis has been the most studied and applied [7,8,9]. This process involves a transesterification reaction in which the hydroxyl groups of the glycols replace the ester groups of the polyurethane, generating polyols that can be reincorporated into the synthesis of new PU foams. These recycled polyols can be fully or partially reused in the formulation of new foams, contributing to the circular economy of the sector and reducing dependence on fossil-based raw materials.

Moreover, the development of more sustainable formulations has led to the replacement of halogenated flame retardants (FRs), widely used in PU foams, with more environmentally friendly alternatives, such as phosphorus-based FRs [10,11,12]. These additives not only reduce the environmental impact and emissions of toxic gases during combustion, but in some cases can also be chemically integrated into the PU matrix, improving the thermal stability of the material without compromising its mechanical properties.

Several studies investigated the inclusion of chemically recycled polyol into PU foam formulations in the last years [9,13,14,15]. Fu et al. [9] studied the glycolysis of flexible PU foam using biobased crude glycerol and then used split-phase recycled polyol to prepare porous hydrophobic sponge and rigid PU foam. The porous sponge had high porosity, a low density of 31.9 kg/m^3^, superwettability, and an excellent selective oil/organic solvent absorption capacity. Meanwhile, the rigid PU showed compressive strength exceeding 150 kPa and met the standards for thermal insulation materials. Chang et al. [13] investigated the optimization of glycolysis using crude glycerol as a solvent for rigid PU foam in the refrigerator sector. This investigation studied the optimization of the polyol recycling process and the production of PU foam with a single-phase recycling polyol, and also conducted a cyclic recycling study on the degradability of recycled PU foam. The regenerated foam showed excellent degradation performance even after multiple recycling cycles. Moreover, a novel flame-retardant PU foam was produced using the obtained recycled polyols. This foam presented a density of 44 kg/m^3^, a compressive strength of 284 kPa, and a thermal conductivity of 0.037 W/m·K. Also, the PU foam achieved improved fire properties, with a limiting oxygen index of 27.8%, and vertical combustion tests achieved a V-0 rating. Aksu et al. [14] studied the production of flexible PU foams using recycling polyol in the range of 10–50 wt% as a substitute of virgin polyol. The results showed that flexible foams of good quality can be obtained with up to 20% by weight of recycled polyol. However, exceeding the optimum content of recycled polyol resulted in a reduction in the air permeability, compression set, and resilience of the resulting flexible PU foams. Wieczorek et al. [15] prepared rigid PU foams replacing up to 50% of virgin polyol with recycled polyol obtained from semi-rigid polyurethane foam waste from the construction sector and also using tris(chloroisopropyl) phosphate (TCPP) as halogen FR (representing 10 wt% of the PU formulation). The PU foam containing 40 wt% recycled polyol maintained a high closed-cell content (up to 87.7%), low thermal conductivity (26.3 mW/mK at 10 °C), and dimensional stability within the limits specified for spray-applied insulation systems (below 1%). In addition, compressive strength improvements of up to 30% were obtained in comparison with the reference foam (294 kPa vs. 208 kPa for reference), mainly due to the increase in density to 48.7 kg/m^3^ for the sample containing 50 wt% recycled polyol. Flammability testing confirmed that all foams, particularly those containing the highest recycled polyol content, complied with the B2 classification requirements according to DIN 4102.

In our previous study [16], the preparation and properties of rigid PU foams made from recycled polyols obtained through the glycolysis of post-industrial rigid PU foam waste from housing and bracket applications were investigated. The partial replacement of commercial polyol with recycled polyols (up to 15 wt%) increased reactivity during foam formation, evidenced by shorter foaming times and more exothermic thermal profiles. The foams obtained showed lower bulk density (88.3–96.9 kg/m^3^) and smaller cells compared to conventional foam. Although the compressive strength decreased slightly, a notable increase in the tensile strength and modulus was observed with 10% recycled polyol, in addition to obtaining a foam with a homogeneous microstructure.

Based on our previous study, the current investigation addresses the production of PU foams using recycled polyol (optimal content of 10 parts by weight with respect to fossil-based polyol) in combination with environmentally friendly phosphorus FRs. The PU formulations were adapted to obtain rigid foams with very low densities around 50 kg/m^3^ for fire-resistant insulation applications. The effect of free-halogen FR is evaluated on the density, cellular structure, thermal conductivity, dimensional and hydrolytic stability, and fire and mechanical properties of the novel foamed material containing recycled polyol. The novelty of this research lies in the use of a recycled raw material obtained from post-industrial rigid PU foam waste from housing and bracket applications to produce high-performance thermal insulation materials with flame-retardant properties. This study therefore explores an alternative feedstock compared to most studies reported in the literature. In addition, the influence of two different types of halogen-free phosphorus-based FRs is investigated: FR900, specifically identified as 3,9-Dimethyl-2,4,8,10-tetraoxa-3,9-diphosphaspiro[5.5]undecane-3,9-dioxide, in powder form with 24% phosphorus content, and reactive polyol-based FR140, an oligomeric ethyl ethylene phosphate, in liquid form with 19% phosphorus content. This investigation contributes to the development of more sustainable PU formulations that integrate both waste recovery and the reduction of the environmental impact associated with the halogen-free FRs.

## 2. Materials and Methods

### 2.1. Raw Materials

For the synthesis of PU foams, a mixture of Lupranol^®^ 3300, Lupranol^®^ 3422, and Lupranol^®^ 3402 from BASF SE (Ludwigshafen, Germany) was used as conventional commercial polyol with a hydroxyl value of 395 mg KOH g^−1^. Polymeric 4,4′-diphenylmethane diisocyanate (pMDI) with an isocyanate (NCO) content of 31% was provided by Arcesso Dynamics S.L (Barcelona, Spain). TEGOAMIN Polycat8 (N,N dimethylcyclohexylamine) from Evonik Nutrition & Care GmbH (Essen, Germany) was used as a gelling and foaming catalyst for the foam formulations without recycled polyol. TEGOSTAB B 8460 (polyether polydimethylsiloxane copolymer stabilizer) from Evonik Nutrition & Care GmbH (Essen, Germany) was used as the surfactant for the foam formulations without recycled polyol. Distilled water was employed as a chemical blowing agent.

The recycled polyol was obtained after an optimized glycolysis reaction of post-industrial rigid PU foams from complex waste for housing and bracket applications in our previous study [17]. The optimized conditions for the glycolysis reaction were 180 °C and 1 h, using ethylene glycol as the glycolysis reactant. The reactants quantities were 1.5 g of solvent with respect to 1 g of PU foam waste, adding 0.002 mol of NaOH catalyst with respect to 1 g of PU foam waste. The final reaction product was further filtered and distilled for solvent removal, resulting in a recycled polyol mixture with a hydroxyl value of 1130 mg KOH g^−1^, viscosity of 10,720 Pa·s at 25 °C, acid value of 4.48 mg KOH g-1, Mn of 885.4 g/mol, and polydispersity index of 1.037.

**Figure 1:**
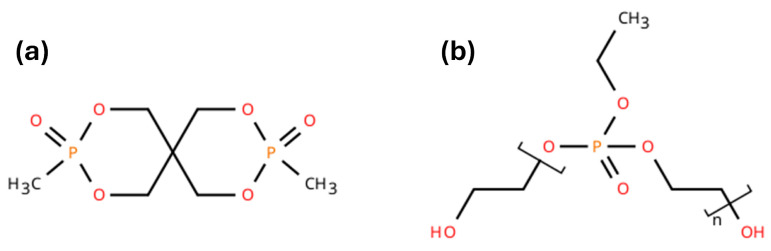
Chemical structure of (**a**) Aflammit PCO-9OO (FR900) [18] and (**b**) AFLAMMIT^®^ PLF 140 (FR140) [19].

Two organic phosphorous-based FRs were used: AFLAMMIT^®^ PCO 900 (FR900) and AFLAMMIT^®^ PLF 140 (FR140) supplied from THOR ESPECIALIDADES SA (Barcelona, Spain). According to the supplier, FR900, specifically identified as 3,9-Dimethyl-2,4,8,10-tetraoxa-3,9-diphosphaspiro[5.5]undecane-3,9-dioxide, is a white powder with a particle size (D99) of 40 microns, a melting point of 245 °C, a processing temperature range between 270 and 280 °C, and a phosphorus content of 24%. FR140, an oligomeric ethyl ethylene phosphate, is a reactive FR with free OH groups that can react with polysiocyanates groups; its viscosity is between 2000 and 4000 mPa·s at 20 °C, and the phosphorus content is 19%. Figure 1 shows the chemical structure of these two FR additives.

### 2.2. Foam Manufacturing

Two series of PU samples were manufactured (Table 1): series 1 with only commercial polyol and series 2 with commercial and recycled polyol. The flame-retardant content of 6 wt% in the total mass of foam was selected according to the literature [20], and previous experimental results are included in the Supplementary Information (Section S1. Study of FR content in small-scale foams). The isocyanate index of 100 is maintained for all formulations. The isocyanate index of 100, defined as the isocyanate/hydroxyl equivalent ratio (NCO/OH), was maintained constant for all formulations. The required amount of isocyanate (parts by weight) was calculated based on the total hydroxyl equivalents present in the PU system, considering the contributions from both the polyols (virgin and recycled polyol) and the reactive flame-retardant FR140. A total mass of 653 g was employed for each formulation [21].

In series 1, a premix of petro-based commercial polyol was prepared with the surfactant, catalyst, and water by stirring with a mechanical mixer at 300 rpm for 3 min. The catalyst content (0.5 ppw) was adjusted to have similar reaction times to that of the foam with recycled polyol (specially around 10 s of cream time for PU_REC). These materials are manufactured as reference systems for comparison with recycled polyol materials. Subsequently, isocyanate was added to the polyol premix and mixed for 8 s at 800 rpm. In series 2, the same foam preparation procedure was followed, but neither catalyst nor surfactant was added as the recycled polyol contained these two components as pointed out in our previous investigation [16]. The recycled polyol content (10 parts by weight with respect to the fossil-based polyol) was also selected based on our previous systematic study [16], in which different substitution levels were evaluated and 10 wt.% was identified as the optimal balance between processing stability and foam performance. After foaming, foamed materials were cured at room temperature for 2 days and specimens were prepared for foam characterization. It should be noted that the amount of catalyst used for the PU system of series 1 (reference without recycled polyol) was adjusted to achieve system reactivity like that of the PU_REC system of series 2 (reference foam containing recycled polyol), which already contained catalysts that had not been removed in the recycling process. The recycled polyol may also contain residual catalyst and surfactant species at very low concentrations (below 1 wt.%), which could not be quantified due to the complexity of the recycled matrix. This aspect could slightly influence the formulation reactivity and should be considered when comparing the different foam systems. Consequently, the comparison between series 1 and series 2 should be interpreted by taking into account these differences, as differences in the catalyst and surfactant content may influence both the foaming behavior and the resulting foam properties. The characteristic times of the reactions for the PU_REC foam (series 2) were 12 s of cream time, 33 s of gel time, 62 of rise time, and 93 s of tack-free time, while for the PU foam (series 1) they were 11 s of cream time, 46 s of gel time, 75 of rise time, and 102 s of tack-free time.

### 2.3. Characterization of Rigid Foams

#### 2.3.1. Foam Density

The apparent density of the foamed materials was measured for three samples with dimensions of 300 mm × 300 mm × 30 mm and examined for each material to calculate the average of density following ASTM D1622-14.

#### 2.3.2. Open Cell Content

The open cell content (OC) was measured by using a gas pycnometer (Accupyc II 1340 from Micromeritics Instrument Corporation (Norcross, GA, USA)), according to ASTM D6226-21: the standard test method for the open cell content of rigid cellular plastics. Three samples of each material (30 × 30 × 30 mm^3^) were analyzed to calculate the average value. OC was calculated by using the following equation:

OC (%)=100 ·((Vsample−Vpycnometer)/(Vsample·p))

where *Vsample* is the geometrical volume of the foamed sample, *Vpycnometer* is the volume measured with the pycnometer, and *p* is the sample porosity calculated using (1− *p_relative_*), in which *p*_relative_ is *p_foam_* (foam density) divided by *p*_solid_ (solid matrix density with value of 1160 kg/m^3^).

#### 2.3.3. Microscopy Characterization

The cellular structure of the foamed materials was observed by scanning electron microscopy (SEM) using Tescan_Vega from Tescan Group, A.S. (Brno, Czech Republic). SEM micrographs were obtained for the growth plane of the foams. The cell diameter was analyzed using image analysis software (ImageJ, v1.48, University of Wisconsin, Madison, WI, USA) using more than 100 cells to calculate the average cell diameter. The cell diameter obtained from 2D micrographs by interactive image analysis was corrected to 3D by multiplying it by a correction factor of 1.273 proposed in the literature [22].

#### 2.3.4. Thermal Conductivity Analysis

Thermal conductivity was determined under steady heat flow conditions using a Rapid K heat flowmeter from HoloMetrix GmbH (Wiesbaden, Germany), in accordance with the ASTM C518-15 standard. The dimensions of the samples were 300 mm × 300 mm × 30 mm (thickness). The measurements were performed at around 25 °C using a temperature gradient of 20 °C. The measurements were carried out 500 days after the foams were produced, to ensure that the carbon dioxide generated during foaming had diffused into the atmosphere and, therefore, the only gas present inside the foam was air [23].

#### 2.3.5. Hydrolytic Stability

Hydrolytic stability was evaluated according to ASTM D2842 (Standard Test Method for Water Absorption of Plastics). Foam samples (80 × 50 × 30 mm^3^) were immersed in distilled water at room temperature for 24 h under controlled conditions. After the water immersion period, the samples were removed, surface-dried with absorbent paper, and weighed. The mass change was calculated as:

$$Mass\;change\;(\%)=\frac{mf-mi}{mi}\;\times \;100$$

where mi is the initial dry mass and mf is the mass after immersion.

#### 2.3.6. Dimensional Stability

Dimensional stability was determined according to ISO 2796 (Cellular plastics, rigid — Test for dimensional stability). Foam samples with dimensions of A × B × thickness (80 × 50 × 30 mm^3^) were measured in the three principal directions using a digital caliper prior from Mitutoyo Europe GmbH (Neuss, Germany) to aging. The samples were then placed in a ventilated oven at 70 °C for 24 h. After exposure, specimens were conditioned at (23 ± 2) °C and remeasured. The dimensional variation was calculated as:

$$\Delta L(\%)=\frac{Lf-Li}{Li}\;\times \;100$$

where Li and Lf represent the initial and final dimensions, respectively. The results are reported as the average of at least three specimens.

#### 2.3.7. Cone Calorimetry Testing

**Figure 2:**
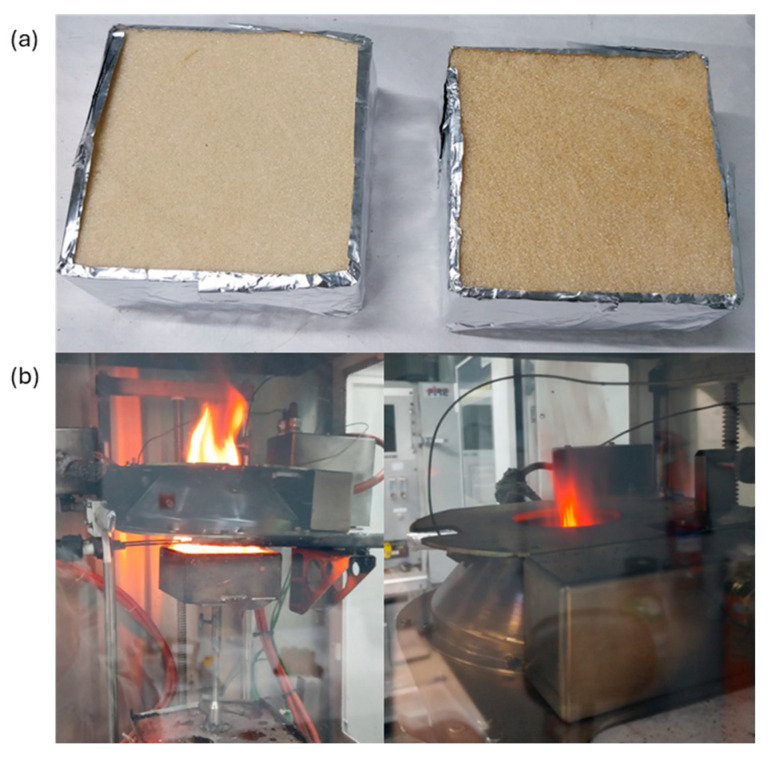
(**a**) Appearance of samples before the cone calorimeter test: (left) PU_REC_6% FR900 and (right) PU_REC_6% FR140; (**b**) cone calorimeter test.

Fire behavior tests were conducted using a fire cone calorimeter system, iCone 2+ from Fire Testing Technology Ltd. (East Grinstead, UK), in accordance with ISO 5660-1:2015 + AMD.1:2019. Specimens measuring 100 mm × 100 mm × 50 mm (thickness) were prepared and exposed to a controlled level of radiation to induce combustion via an external spark (Figure 2a). Test conditions involved a radiant heat flux value of 25 kW/m^2^ on the specimen’s surface. The distance between the radiant heater and the specimen surface was 25 mm. To determine the heat release rate (HRR), oxygen consumption measurements were taken by collecting data on the exhaust duct flow rate and oxygen concentration at 2 s intervals. The HRR data were then used to determine the q_max_ or HRR_max_ (Maximum Heat Release Rate), as well as the Average Rate of Heat Emission (ARHE) curve, whose highest value corresponds to the Maximum Average Rate of Heat Emission (MARHE) parameter. The average Mass Loss Rate (MLR) was calculated by dividing the total mass lost by the specimen over the test duration by the exposed surface area of the specimen. The Total Mass Loss Rate (TMLR) measures the rate at which a material loses mass over time during combustion. The cone calorimeter equipment is shown in Figure 2b.

#### 2.3.8. Mechanical Properties Testing

Mechanical properties in compression were measured at room temperature using an MTS Alliance RF/150 Machine, according to ISO 844:2021. Stress–strain curves were obtained at a strain rate of 3 mm/min. The compression tests were carried out in the growing direction as this is the direction of interest since the potential applications of the foamed materials are as core sandwich panels for the building sector. At least five samples with dimensions of 40 mm × 40 mm × 30 mm (thickness) were measured for each material. Young’s modulus and compressive strength were calculated from the stress–strain curves.

## 3. Results

### 3.1. Characterization of PU Foams

**Figure 3:**
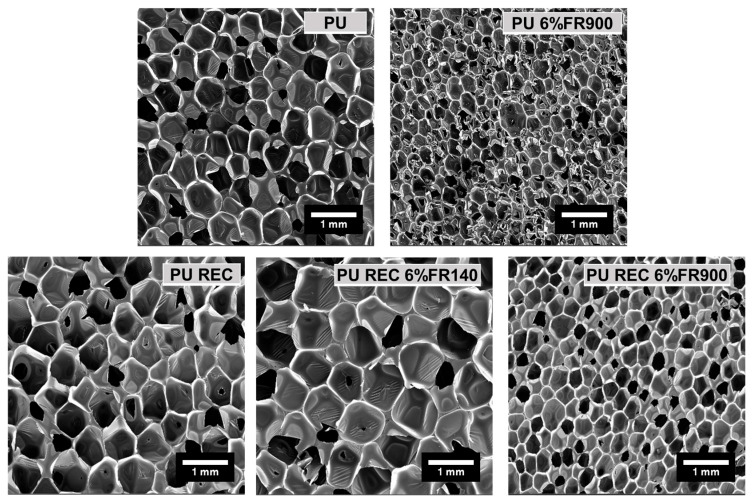
SEM images of foamed materials.

The density and cellular structure parameters of foamed materials under study were characterized to understand their final properties (Table 2 and Figure 3). Moreover, the thermal conductivity was measured after 500 days of foam manufacturing to ensure that the exchange of gases from the cells to the outside ended and was thus stable (Table 2). In the case of foams containing only commercial polyol (PU and PU 6%FR900), the addition of FR900 maintained the values of density and cell content with respect to the reference material. A nucleating effect was observed when FR900 was added to the PU formulation, reducing the cell size from 689 to 433 microns. The distribution of cell sizes was slightly more heterogeneous, with an NSD value of 0.32 compared to 0.28 for the reference material, and the cell anisotropy slightly increased, although it remained within a similar range for all formulations, indicating no significant effect of this parameter on thermal conductivity. Regarding the thermal conductivity performance, the foamed material FR900 reached the lowest value of 36.10 mW/mK, which was mainly related to the significant decrease in cell size (37%) that reduced the radiative term of total thermal conductivity [24].

For the foams containing both commercial and recycled polyol, the inclusion of recycled polyol caused a decrease in density from 50 kg/m^3^ to 44.1 kg/m^3^ compared to the PU reference without recycled polyol. This result may be related to the high reactivity of the reaction mixture containing recycled polyol, which contains an excess amount of catalyst from the recycling process. Furthermore, this high reactivity of the reaction mixture made the growth of the cell structure more unstable, with a larger cell size and a small increase in the open cell content compared to the PU sample without recycled polyol. The thermal conductivity of the reference material containing recycled polyol was maintained with respect to the reference PU without recycled material (38.73 mW/mK for PU_REC vs. 38.67 mW/mk for PU). Despite its high decrease of 6 kg/m^3^, which reduced heat conduction through the solid phase [24], it increased in cell size, thus increasing the radiative term. When analyzing the inclusion of both FRs, FR900 and FR140, in the recycled polyol formulation, significant changes in the final characteristics of the foams could be observed. FR140, particularly OH-terminated phosphorus polyol-based FR, is a reactive FR that produces an increase in foam density (53 kg/m^3^), open cell content (~22%), and cell size (1041 microns). This FR140 is added in liquid form and, as it has a very low viscosity (2000–4000 mPa·s at 20 °C), it reduces the viscosity of the reaction mixture, which can promote cell degeneration mechanisms. This deterioration of the cell structure leads to a slight increase in the open cell content and cell size compared to foam without recycled polyol. Therefore, the foamed material containing FR140 exhibits the greatest increase in thermal conductivity (40.92 mW/mK), due to the larger cell size and higher density that increase the radiative contribution and conduction through the solid phase of the thermal conductivity, respectively.

On the other hand, FR900, in a solid form, also increases the density of the foam and the open cell content. In this case, the use of a solid FR increases the viscosity of the reaction mixture, which generates a lower expansion effect on the material, reaching around 50 kg/m^3^. In addition, this FR significantly affected the open cell content, reaching 72%, which could be due to the large size of FR (40 microns) that could break the structure of the cells that may be more unstable when recycled polyol is used compared to material without recycled polyol (PU_6%FR900). But, this FR has a nucleating effect, as already observed in foam without recycled polyol, which reduced the cell size by almost 50% compared to its reference (465 microns for PU_REC_6%FR900 vs. 848 microns for PU_REC). Despite its high open cell content, this material, made with recycled polyol and 6%FR900, has a low thermal conductivity value of 37.04 mW/mK. Although a high open cell content is generally expected to increase the thermal conductivity due to greater conduction through the gas phase, this effect can be considered negligible in the present study. This is because the thermal conductivity was measured in the aged state, after all the carbon dioxide from the blowing agent had been diffused out of the foam cells and had been replaced by atmospheric air. Under these conditions, the conduction through the gas phase contribution becomes independent of the degree of cell opening [23]. Therefore, the overall heat transfer was mainly determined by radiative contribution, which is strongly dependent on cell size. Consequently, the significant reduction in cell size induced by FR900 effectively decreased radiative heat transfer, which explains the relatively low thermal conductivity despite the high open cell content [24]. It is interesting to note that the aged thermal conductivity values obtained in this study are within the typical range reported for commercial water-blown rigid PU insulation foams with densities in the range of 40–60 kg/m^3^. In such materials, aged thermal conductivity values usually range from 34 to 40 mW/mK once the cell gas composition approaches air equilibrium [3,24,25]. Although slight differences between formulations were observed, their magnitude was relatively small and comparable to the experimental uncertainty and to the values reported for commercial materials. The differences in thermal conductivity between foamed systems were confirmed to be statistically significant by one-way ANOVA analysis (*p* < 0.05), supporting the reliability of the observed trends.

In addition, the dimensional and hydrolytic stability of the PU foams were measured (Table 3). The dimensional stability results at 70 °C show that all PU formulations have relatively low dimensional variations in the three dimensions, indicating good thermal stability under the tested conditions. The reference PU and PU_6%FR900 foams maintained very stable dimensions, with only minor changes that could be attributed to thermal expansion. In the PU foams containing recycled polyol, PU_REC showed a slightly higher variation in thickness. The incorporation of 6% FR140 and FR900 improved dimensional stability in the recycled system, leading to minimal dimensional changes.

Regarding hydrolytic stability, water uptake strongly correlates with the degree of open cell content. PU foams with predominantly closed-cell structures presented a low mass increase after immersion. The PU foams containing recycled polyol and FRs had higher water absorption values, particularly PU_REC_6%FR900, which displayed the highest uptake due to its highly open cellular structure (73% of open cell content) [26].

In general, the results obtained in this section indicate that the use of recycled polyol and the type of FRs influence the density, foam microstructure, and resulting properties of the foamed materials. From a processing perspective, FR incorporation can also affect the rheology of the reactive mixture, which is a key parameter for foam formation and industrial processability. In particular, the use of the solid FR900 requires adequate dispersion within the polyol phase to ensure a homogeneous reactive mixture and, consequently, consistent final properties. Poor dispersion can lead to particle agglomeration, which can cause heterogeneities and the deterioration of the cellular structure of the foams [27]. In addition, the inclusion of solid FRs such as FR900 is expected to increase the viscosity of the reactive mixture due to a filler effect and filler–matrix interactions, affecting the foaming process [28]. On the other hand, the incorporation of the liquid reactive FR140 facilitates a more homogeneous distribution within the polyol phase due to its liquid nature, reducing potential dispersion-related issues compared to solid FR additives [29]. At the same time, it can modify both the viscosity and the chemical reactivity of the PU system. Due to its reactive nature, FR140 contributes to the hydroxyl functionality of the formulation and may influence the balance between blowing and gelation reactions [30], which is a critical factor in foam formation and structure development. In this study, this behavior is reflected in the increase in cell size and a moderate increase in foam density, while only a small increase in the open cell content is observed, suggesting that FR140 mainly affects cell growth rather than cell opening. These aspects will be considered in future work addressing formulation scalability. Moreover, the environmental benefits of the foams produced using recycled polyol are further supported by the Life Cycle Assessment (LCA) results provided in the Supporting Information (Section S2).

### 3.2. Fire-Retardant Properties

**Figure 4:**
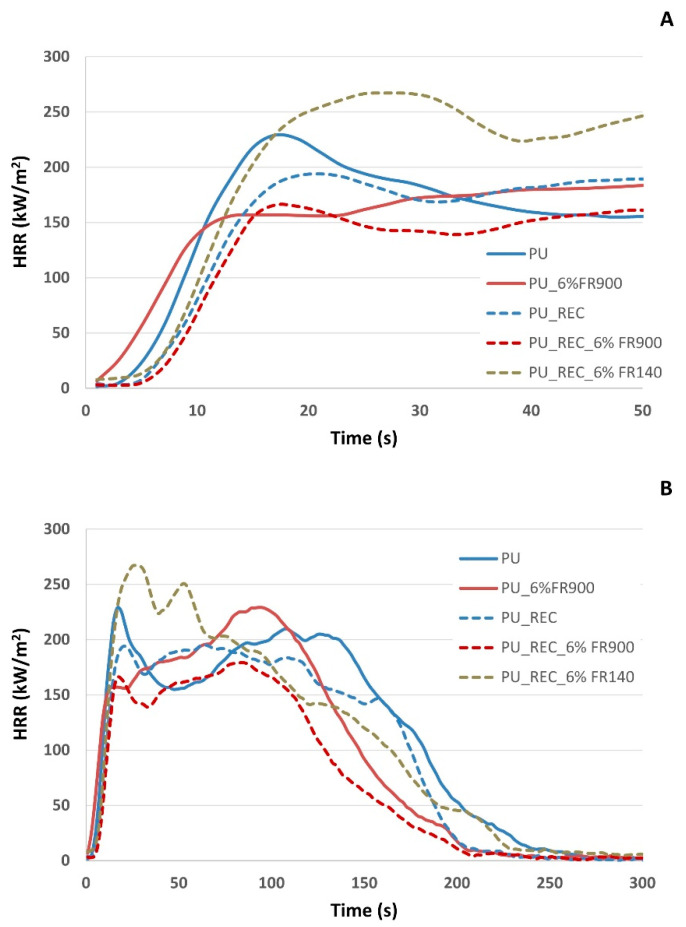
Heat Release Rate (HRR) curves for foamed materials without recycled polyol and with recycled polyol: (**A**) zoomed-in image of HRR curves in the first seconds of the cone calorimetry test, and (**B**) HRR curves at 300 s of the cone calorimetry test.

The fire behavior was characterized by cone calorimetry, considering different formulations that varied in their content of recycled polyol (replaced 10% of the virgin polyol) and FR additives (6 wt%). The results obtained in the cone calorimeter test are presented in Table 4. Moreover, HRR curves are shown in Figure 4. In the series of formulations prepared without recycled polyol, the un-fire-retardant material was compared to the same material treated with the FR900 additive. In this instance, the overall cone calorimeter results are not drastically different. The average MARHE value was slightly higher in the formulation containing the FR900 fire retardant. However, it was observed that the fire retardant led to a more uniform HRR curve and an improved time to extinction (reduced by 25 s).

In the formulations containing recycled polyol, the inclusion of recycled polyol in the formulations did not have an unfavorable effect on fire performance, since the measured fire parameters are lower than those of the reference without recycled polyol (Figure 4 and Table 4). The sample treated with FR140 exhibits the highest values for MARHE (kW/m^2^), THR 1200 s (MJ/m^2^), and q.max (kW/m^2^), suggesting it has the worst fire performance. Conversely, the formulation treated with the FR900 additive shows the lowest values for the fire parameters (MARHE, q.max, THR, MLR, and TMLR), indicating the best fire performance. The untreated reference formulation falls in an intermediate position between the two recycled polyol formulations with FRs. For all formulations, the time to ignition remains virtually the same. However, the time to extinction varies, ranging from 197 s for the recycled polyol formulation treated with FR900 to 244 s for the untreated reference formulation made with virgin polyol. In the formulation treated with the FR140 additive, its fire performance is worse than that of the untreated formulations. Regarding the maximum deformation during the test, no changes were observed in any of the samples under study. The poor fire properties of the FR140 system relative to foam containing FR900 could be related to the lower phosphorus content in the FR additive (19% for FR140 vs. 24% for FR900) and also to the significantly large cell morphology (1041 micros for FR140 foam and 465 microns for FR900 foam). This last result indicates a lower FR distribution around the solid phase, which can hinder the formation of a protective char layer. From a fire mechanistic point of view, FR140, an oligomeric ethyl ethylene phosphate with hydroxyl functionality, is chemically incorporated into the PU network through a reaction with isocyanate groups. During thermal degradation, phosphorus-containing species can promote char formation in the condensed phase via dehydration and crosslinking reactions, leading to the development of a carbonaceous layer that acts as a barrier to heat and mass transfer [31]. In addition, a secondary contribution in the gas phase may arise from the release of phosphorus-containing radicals (e.g., PO·), which can interfere with flame propagation by scavenging H· and OH· radicals [31]. However, in the present system, these flame-retardant mechanisms did not result in improved fire performance. This behavior can be attributed not only to the lower phosphorus content, but also to the dominant effect of cellular structure. In particular, the larger cell size observed for FR140 foams may enhance heat transfer and facilitate the release of combustible volatiles, thereby counteracting its potential flame-retardant action. Conversely, FR900, by providing a higher phosphorus content and generating a smaller open cell structure, promotes a more homogeneous and effective carbonaceous barrier [32,33]. The synergy between chemical composition and cellular microstructure is critical for stabilizing solid residues and limiting the release of volatiles [34]. Moreover, despite the increase in density, the improved fire performance indicates that cellular structure plays a more relevant role than foam density under the studied conditions. On the other hand, foams based on recycled polyols provide an intrinsic FR effect due to the presence of more aromatic segments originating from the original isocyanate structure; this promote dense carbonization and shift thermal degradation toward higher temperatures [35].

**Figure 5:**
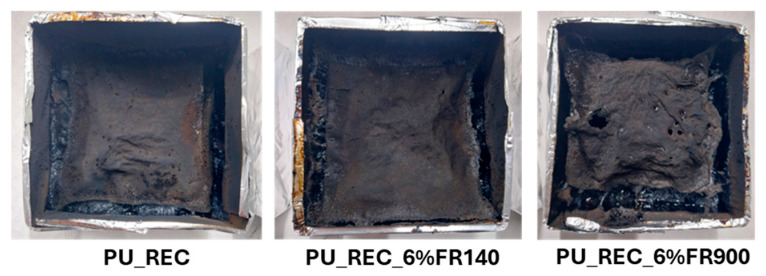
Appearance of samples after the cone calorimeter test for PU foams with recycled polyol: PU_REC, PU_REC_6% FR900, and PU_REC_6% FR140.

In addition, the appearance of the residues after the cone calorimeter test for foams with recycled polyol is shown in Figure 5. PU_REC and PU_REC_FR900 show relatively compact and continuous char layers covering the surface of the specimen, indicating the formation of a stable carbonized structure during combustion. These char layers remain mostly intact and homogeneous, suggesting an effective barrier effect that may limit heat and mass transfer during burning. This behavior is consistent with their more homogeneous cellular structure, which favors the formation of a continuous protective layer. On the contrary, PU_REC_FR140 presents a noticeably different char morphology, characterized by a more expanded and porous residue with visible holes and a less cohesive structure. This more fragile char indicates the lower structural integrity of the protective layer formed during combustion, which may reduce its ability to protect the underlying material from heat and oxygen. This weaker char structure is also consistent with the larger cell size and less stable cellular morphology of this formulation. The fire performance assessment in this investigation was based on cone calorimetry results; however, additional tests, such as limiting oxygen index (LOI), UL-94 classification, smoke density, and toxic gas analysis, will be considered in future work in order to provide a more comprehensive understanding of the fire behavior of foamed systems.

### 3.3. Mechanical Properties

To complete the investigation, the mechanical properties were evaluated for the foamed materials under study by compression testing. To compare foams with different densities, the scaling relationship between mechanical properties and density [35] was employed to exclude the effect of density on the mechanical properties. Table 5 presents the relative compressive strength and relative Young’s modulus results (Young’s modulus and compressive strength divided by the density of the foam). The foamed samples with virgin polyol present better mechanical properties in comparison with the foamed samples containing recycled polyol, due to their relatively high density and low open cell content. In particular, the incorporation of FR900 additives leads to a significant increase in Young’s modulus and compressive strength values, which can be attributed to the formation of a finer cellular structure, as evidenced by the reduction in cell size from 689 to 433 µm.

In contrast, the incorporation of recycled polyol results in a marked decrease in mechanical properties. This behavior is mainly associated with the changes in the cellular structure, particularly the increase in open cell content. PU_REC already shows slightly higher open cell values compared with the virgin formulation, which contributes to the reduction in stiffness and collapse strength. When FRs are introduced into the recycled formulations, the mechanical performance becomes strongly dependent on the resulting foam morphology. In particular, PU_REC_6%FR900 has a very high open cell content (~73%), which significantly weakens the cellular structure despite its relatively small cell size. Similarly, the formulation containing FR140 shows increased cell size and open cell content values, both of which contribute to the observed reduction in mechanical performance. It is worth noting that the differences observed in the mechanical properties were statistically significant, as confirmed by one-way ANOVA analysis (*p* < 0.05).

It should be noted that the values obtained for these series of foamed materials are within or above the range of the collapsed stress requirements for low-density rigid PU insulation panels used in building applications, specifically in the range of 0.10–0.20 MPa at 10% deformation according to EN 13165:2012+A2:2016 standard.

## 4. Conclusions

In this study, more sustainable water-blown rigid polyurethane (PU) foams were investigated using recycled polyol and halogen-free flame retardants (FRs) for potential fire-resistant insulation applications. The replacement of 10% virgin polyol enabled the obtention of foamed material with a thermal insulation performance comparable to conventional systems. In particular, the formulation containing recycled polyol and 6 wt% FR900 (PU_REC_6%FR900) showed the best overall performance. Remarkably, this material with a high open cell content of 72% showed a lower thermal conductivity value of 37.04 mW/mK, corresponding to around 4% reduction with respect to the reference materials. This reduction is mainly attributed to the significant reduction in cell size (465 microns), reducing the radiative term of thermal conductivity. Regarding fire properties, the FR900 additive notably improved cone calorimeter parameters, such as reducing the peak and total heat release with respect to the rest of the foamed samples (e.g., 197 s of extinction time, 148.2 kW/m^2^ of Maximum Average Rate of Heat Emission, and 24.6 MJ/m^2^ of Total Heat Release), while the FR140 additive worsened fire behavior. Moreover, the FR900 additive improved the mechanical properties in the compression of the foamed system containing recycled polyol (e.g., 6.97 MPa of Young’s modulus and 0.24 MPa of compressive strength). In this study, a more sustainable insulation material with good fire-retardant and mechanical performances was developed, including recycled polyol and a halogen-free retardant in its composition. The obtained results highlight the potential of these formulations for industrial-scale polyurethane foam production for the construction industry while contributing to reduced environmental impacts.

## Figures and Tables

**Table 1:** Foam formulations of the different materials.

Series	Reference	Commercial Polyol (ppw)	Recycled Polyol (ppw)	Water (ppw)	Surfactant (ppw)	Catalyst (ppw)	Isocianate (ppw)
1	PU	100	0	2	1	0.5	138
	PU_6%FR900	100	0	2	1	0.5	138
2	PU_REC	90	10	2	0	0	158
	PU_REC_6%FR140	90	10	2	0	0	158
	PU_REC_6%FR900	90	10	2	0	0	158

**Table 2:** Density, open cell content, cell diameter, normalized standard deviation (NSD) cell anisotropy, and thermal conductivity of foamed materials.

Foam Reference	Density (Kg/m^3^)	Open Cell Content (%)	Cell Size 3D (µm)	NSD (SD/Cell Diameter)	Cell Anisotropy	Thermal Conductivity (mW/mK)
PU	50.0 ± 0.7	13.70 ±3.94	689 ± 149	0.28	1.14 ± 0.22	38.67 ± 0.05
PU_6%FR900	49.8 ± 0.6	11.68 ± 0.17	433 ± 109	0.32	1.30 ± 0.25	36.10 ± 0.06
PU_REC	44.1 ± 0.8	15.09 ± 0.64	848 ± 191	0.29	1.24 ± 0.24	38.73 ± 0.04
PU_REC_6%FR140	52.7 ± 0.4	21.71 ± 1.09	1041 ± 228	0.28	1.13 ± 0.18	40.92 ± 0.08
PU_REC_6%FR900	50.3 ± 0.9	72.86 ± 5.72	465 ± 92	0.25	1.29 ± 0.30	37.04 ± 0.09

**Table 3:** Dimensional variations at 70 °C for 24 h and mass change after water immersion of foamed materials.

	Dimensional Stability at 70 °C for 24 h			Hydrolytic Stability for 24 h
Foam Reference	Change in Side A, ΔL (%)	Change in Side B, ΔL (%)	Change in Thickness, ΔL (%)	Mass Change (%)
PU	0.80 ± 0.14	0.69 ± 0.63	0.36 ± 0.11	12.80 ± 1.62
PU_6%FR900	0.42 ± 0.23	0.15 ± 0.29	0.45 ± 0.12	11.39 ± 2.49
PU_REC	0.50 ± 0.63	0.21 ± 0.59	1.56 ± 0.01	12.90 ± 3.38
PU_REC_6%FR140	0.06 ± 0.07	−0.01 ± 0.10	0.22 ± 0.57	13.89 ± 2.13
PU_REC_6%FR900	−0.70 ± 0.39	−0.20 ± 0.17	0.47 ± 0.19	35.13 ± 1.46

**Table 4:** Cone calorimeter test results of foamed materials: ignition time, extinction time, Maximum Average Rate of Heat Emission (MARHE), Total Heat Release (THR) at 1200 s, Maximum Heat Release Rate (q.max or HRR max), Average Mass Loss Rate (MLR), Total Mass Loss Rate (TMLR) at 1200 s, and maximum deformation of the samples during the test.

Reference	Ignition Time (s)	Extinction Time (s)	MARHE (kW/m^2^)	q.Max or HRR Max (kW/m^2^)	THR 1200 s (MJ/m^2^)	MLR (g/m^2^⋅s)	TMLR 1200 s (g/m^2^)	Maximum Deformation During Test (mm)
PU	14	244	176.7	238.5	35.1	1.574	1882.0	0
PU_6%FR900	15	219	180.3	226.7	30.5	1.673	1993.9	0
PU_REC	15	201	170.4	195.8	29.8	1.318	1561.9	0
PU_REC_6%FR140	15	229	213.0	269.8	37.5	1.656	1993.6	0
PU_REC_6%FR900	15	197	148.2	179.1	24.6	1.331	1579.3	0

**Table 5:** Mechanical properties in the compression test for the foamed samples.

Reference	Density (Kg/m^3^)	Young’s Modulus (MPa)	Compressive Strength (MPa)	Relative Young’s Modulus (MPa)	Relative Compressive Strength (MPa)
PU	50.0 ± 0.7	13.52 ± 1.89	0.40 ± 0.01	0.270	0.008
PU_6%FR900	49.8 ± 0.6	17.75 ± 0.72	0.54 ± 0.04	0.356	0.011
PU_REC	44.1 ± 0.8	4.58 ± 0.47	0.18 ± 0.03	0.104	0.004
PU_REC_6%FR140	52.7 ± 0.4	6.60 ± 0.66	0.28 ± 0.03	0.125	0.005
PU_REC_6%FR900	50.3 ± 0.9	6.97 ± 0.66	0.24 ± 0.01	0.139	0.005

## Data Availability

The original contributions presented in this study are included in the article. Further inquiries can be directed to the corresponding author.

## Supplementary Materials

The following supporting information can be downloaded at: https://www.mdpi.com/article/10.3390/polym18070856/s1, Figure S1: Two series of small-scale PU foams incorporating different FRs, FR140 and FR900, at contents of 0, 3, 6, and 10 wt.%; Figure S2: Density of the two series of small-scale PU foams incorporating different FRs, FR140 and FR900, at contents of 0, 3, 6, and 10 wt.%; Figure S3: SEM micrographs of the two series of small-scale PU foams incorporating different FRs, FR140 and FR900, at contents of 0, 3, 6, and 10 wt.%.
