# Supplementary material for: Impact of Eco-Friendly Flame-Retardant Water-Blown Rigid Polyurethane Foams Containing Recycled Polyols for Insulation Applications
Source: Polymers (Basel). 2026 Mar 31;18(7):856. doi: 10.3390/polym18070856 (PMC13074954; doi:10.3390/polym18070856)
Supplement: Supplementary file 1 [file polymers-18-00856-s001.zip › polymers-4155997-supplementary.pdf]

polymers

MDPI

Supporting Information

1. Study of FR contents in small scale foams

A series of small-scale rigid polyurethane (PU) foams was prepared incorporating two different flame retardants (FRs), FR140 and FR900, at contents of 0, 3, 6, and 10 wt.% relative to the total mass of the foam. The different series of PU foams were manufactured following the formulations reported in **Table 1** of the manuscript and using a total mass of 40 grams. The foams were produced at small-scale using plastic containers (1L) as molds to evaluate the influence of FR contents (**Figure S1**). The small-scale foams were visually examined after curing, with particular attention paid to the top surface (foam cup surface), as this area is especially sensitive to gas evolution and cell stability during the foaming process. It was observed that PU formulation containing 10 wt.% FR900 showed the presence of surface holes and irregularities on the top surface. These surface defects are attributed to gas loss during the final foam expansion [1], while lower FR contents showed a more homogeneous and uniform surface morphology. On the other hand, foams containing FR140 presented a rougher foam surface, although without the presence of surface perforations or gas release defects.

In addition, the density and cellular structure of the PU foams were characterized to evaluate the influence of FR incorporation on the foam expansion and morphology (**Figures S2** and **S3**). Density measurements showed that foams containing 10 wt.% FR, including FR900 and FR140, present an increase in apparent density compared to the reference formulation (**Figure S2**). This behavior indicates that higher FR contents affect the foaming process, with also influences cell growth and overall expansion efficiency. In addition, PU foams containing FR900 presented higher density values than those containing FR140 at equivalent contents. This difference can be attributed to the physical nature of the FRs: FR900, supplied in solid form, may act as a particulate filler and partially hinder foam expansion, whereas FR140, being a liquid polyol-based FR, is more homogeneously incorporated into the reactive system and applies a comparatively lower restriction on cell expansion.

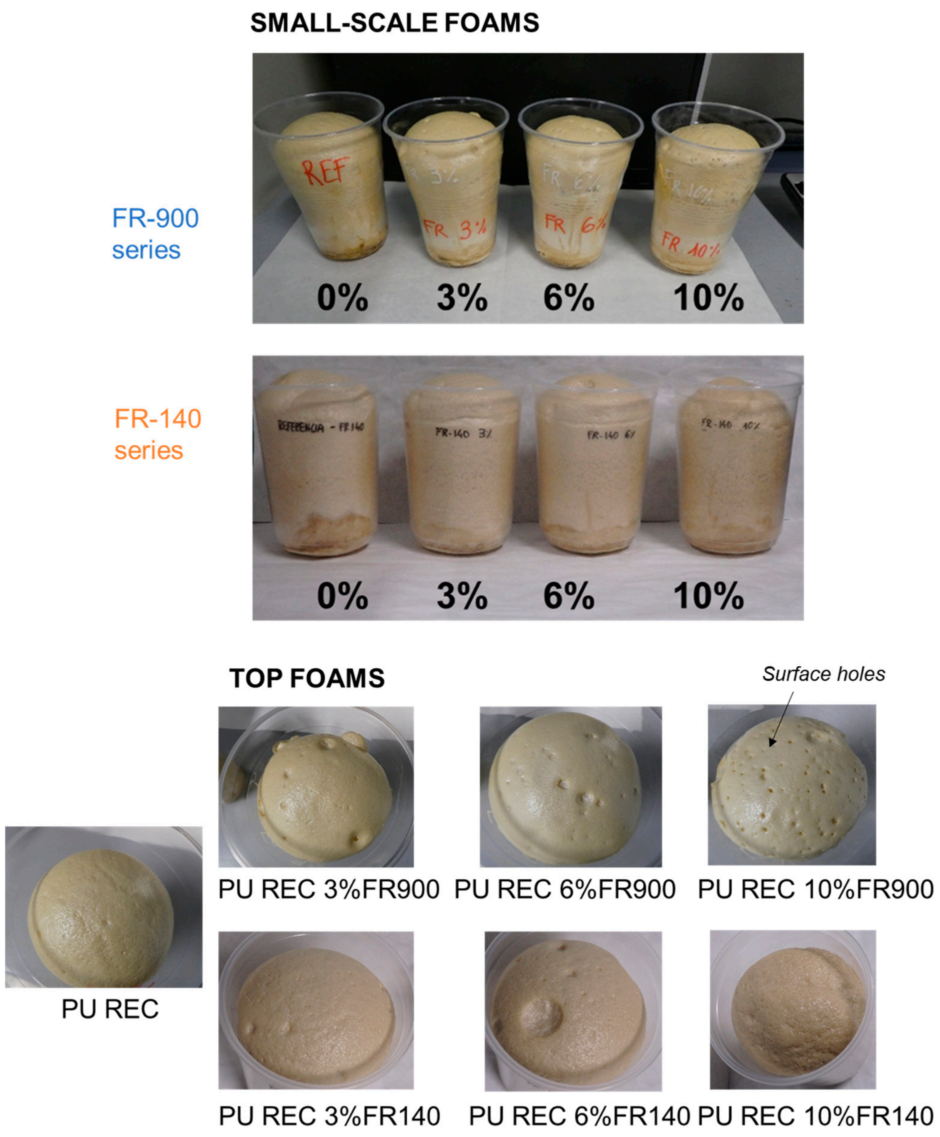

Figure S1. Two series of small-scale PU foams incorporating different FRs, FR140 and FR900, at contents of 0, 3, 6, and 10 wt.%.

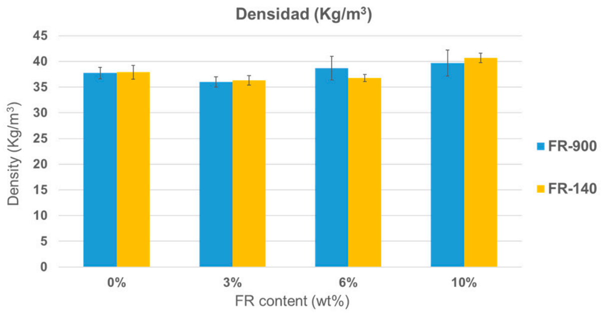

Figure S2. Density of the two series of small-scale PU foams incorporating different FRs, FR140 and FR900, at contents of 0, 3, 6, and 10 wt.%.

To further clarify these observations, SEM characterization was performed to examine the cellular morphology of the PU foams (**Figure S3**). For the FR900 series, the average cell size remained relatively stable with increasing FR content, indicating that FR900 did not significantly alter cell nucleation or growth within the investigated range. In contrast, the FR140 series showed an increase in cell size at 6 wt.% and 10 wt.% contents, suggesting that higher concentrations of the liquid reactive FR may affect the balance between blowing and gelation reactions, leading to slight cell degeneration mechanisms.

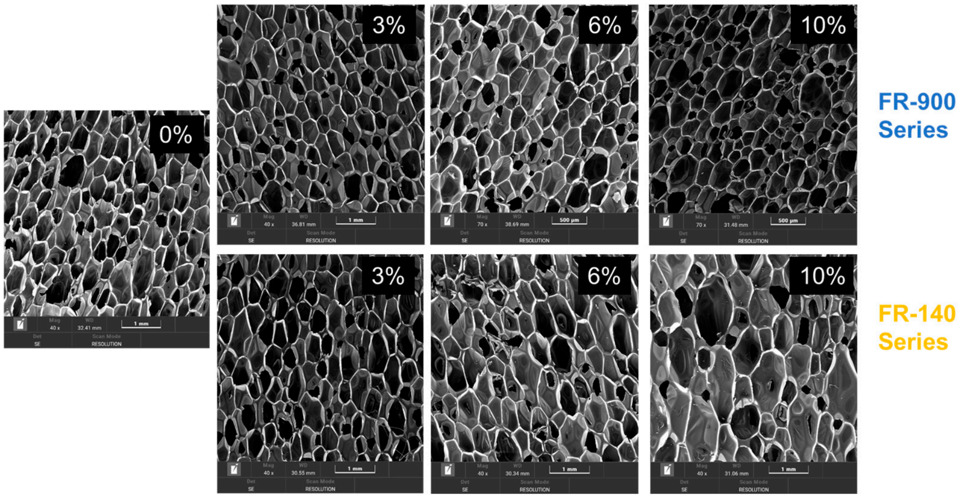

Figure S3. SEM micrographs of the two series of small-scale PU foams incorporating different FRs, FR140 and FR900, at contents of 0, 3, 6, and 10 wt.%.

Considering the combined results from visual inspection, density analysis, and SEM characterization, a FR content of 6 wt.% appears to provide an optimal balance between structural integrity and formulation stability for both systems. Thus, this optimum FR percentage was selected as the starting point for conducting this study, coinciding with the percentage selected in the literature [2].

2. Life Cycle Assessment (LCA)

LCA of the PU foam from recycled polyol was carried out to evaluate its reduced CO_2 footprint compared to a fossil-based PU foam (baseline) with similar characteristics. Life Cycle Assessment (LCA) was carried out according to ISO 14040 (Environmental management — Life cycle assessment — Principles and framework) and ISO 14044:2006 (Environmental management — Life cycle assessment — Requirements and guidelines), using GaBi software developed by Sphera. For the LCA baseline calculation it was used GaBi Software (Sphera) database. For this study, it will be assumed that the foam sheets are installed on the walls of a small 9 m² building. The environmental impact was calculated in production stage, transport stage to the implementation site (100 km), use stage (1 year of used) and end of life (recycling). The functional unit was defined as 30 m² of insulation sheet of foam composed of fossil polyol or recycled polyol.

Inventory list has been completed in all stages:

Production stage: In this Stage CIDAUT only include the raw materials supply and the manufacture of the foams.

Emissions: 20.2 kg CO_2 eq for foam with recycled polyol versus 303 kg CO_2 eq for fossil foam.

These emissions are mainly associated with the isocyanate used in foam production and the percentage of fossil-based polyol production. In the case of fossil polyol foam (conventional system), it consumes 303 kg CO_2 due to the higher use of fossil-derived feedstock.

Transport stage: It is estimated that the foam travels 100 km to the installation site of the materials.

Emissions (100 km of transport): 3.26 kg CO_2 eq for both types of foams.

The transport emissions are the same for both products (polyol and recycled polyol-based foam).

Use stage: The recycled polyol foam reduces the energy consumption by 7% (lower thermal conductivity value). The energy consumption of fossil polyol foam in 1 year is 5529,6 MJ.

Emissions: 578 kg CO_2 eq for foam with recycled polyol versus 631.04 kg CO_2 eq for fossil foam.

Recycling stage: The foams are recycled by chemical recycling process (glycolysis). The recycled polyol is reintroduced into the cycle, creating a closed loop. For this reason, the production stage generates less impact because it needs to produce less quantity of initial product.

Emissions: 222 kg CO_2 eq for both types of foams.

Total environmental impacts for foam containing recycled polyol and conventional foam containing fossil polyol:

Total emissions of recycled polyol foam: 823 kg CO_2 eq.

Total emissions of fossil polyol foam (100% fossil origin): 1159.3 kg CO_2 eq.

References

1. Gibson LJ (2003) Cellular Solids. MRS Bull 28:270–274. https://doi.org/10.1557/mrs2003.79
2. Zemła M, Prociak A, Michałowski S (2021) Bio-Based Rigid Polyurethane Foams Modified with Phosphorus Flame Retardants. Polymers (Basel) 14:102. https://doi.org/10.3390/polym14010102
